# Feline lymphoma of the nervous system. Immunophenotype and anatomical patterns in 24 cases

**DOI:** 10.3389/fvets.2022.959466

**Published:** 2022-09-08

**Authors:** Maria Teresa Mandara, Alessia Domini, Giuseppe Giglia

**Affiliations:** Neuropathology Laboratory, Department of Veterinary Medicine (DVM), University of Perugia, Perugia, Italy

**Keywords:** anatomical pattern, cat, immunophenotype, lymphoma, nervous system

## Abstract

This study aimed to describe the specific localization and anatomical pattern of 24 feline lymphomas of the nervous system for which the immunophenotype was identified by immunohistochemistry investigations to support the potential specific correlation between subtypes and anatomical patterns. In total 10 tumors affected the spinal cord, eight the brain, four the peripheral nerves, one involved both the brain and the spinal cord, and one simultaneously the brain and the optic nerves. Twenty two tumors were primary lymphomas. The affected animals were 8 years of mean age. Tumors developed as an extra-axial mass (11 cases), intra-axial mass (six cases), leptomeningeal lymphomatosis (three cases), and neurolymphomatosis (five cases). One of them expressed both leptomeningeal lymphomatosis and neurolymphomatosis patterns. Two intra-axial brain lymphomas showed an angiotropic pattern. The optic chiasm was the most involved site for neurolymphomatosis. Immunolabeling was performed using anti-CD3, CD20, CD79a, PAX5, MUM-1, CD56, and anti-CD44 antibodies. In total, 12 tumors consisted of B cell lymphomas, and six of T cell lymphomas, two cases were double-reactive lymphomas while two cases consisted of non-B non-T lymphomas. B cell lymphoma affected animals of 6.4 years of mean age, while the T cell lymphoma affected older animals (mean age of 11.1 years). Extra-axial tumors mainly consisted of B cell lymphomas (8/11). Neurolymphomatosis expressed different immunophenotypes, and the B cell phenotype was the most prevalent in the optic chiasm. Two leptomeningeal lymphomatoses expressed T cell immunophenotype. For the first time, plasmacytoid differentiation was found for angiotropic lymphoma and neurolymphomatosis. All the cases, except one, were CD56-negative. CD44-expression confirmed a common malignant potential for all the anatomical patterns of the nervous system lymphoma in cats. Immunophenotype of feline lymphoma of the nervous system and its potential association with specific anatomical patterns should be strongly required in the diagnostic workup and clinical approach to this tumor especially when its primary origin is confirmed.

## Introduction

Lymphoma represents the most common hematopoietic disorder in cats, accounting for up to 90% of tumors of this system (1). In addition, it represents the most common neoplasia in young cats (2). In contrast to its canine counterpart, in the cat, the solitary lymphoma is overall more common than the multicentric form, and the gastrointestinal anatomical pattern is assumed as the most frequent (3). Not infrequently, the solitary form develops in the nasal/paranasal site, the kidney, and the nervous system (4).

In the feline specie, lymphoma of the nervous system accounts for no more than 13% of all lymphomas, most commonly as a secondary event in a multicentric neoplastic disease (5, 6). In recent years, a not negligible incidence of 24% has been reported for the primary feline lymphoma occurring in the central nervous system (7). Although the overall prevalence of lymphoma affecting the nervous system is rather limited, in the cat, this tumor represents the most common intracranial neoplasia (14.4%) after meningioma (58.1%) (8), and the first tumor of the spinal cord with 38.8%, followed by vertebral osteosarcoma (16.5%) (9). For these reasons, lymphoma of the nervous system should be placed in the group of major feline neurological diseases (10).

Neuropathology and advanced imaging experienced in the last thirty years have shown that lymphoma of the nervous system can occur with a wide range of anatomical patterns. In the cranial cavity, feline lymphoma can develop as intraparenchymal (intra-axial) or, not infrequently, extraparenchymal (extra-axial) lymphoma (5). In about 50% of cases, the intra-axial brain lymphoma grows as a lymphomatosis cerebri consisting of a diffuse parenchymal infiltration (8). Although less common, additional anatomical patterns of the feline nervous system lymphoma consist of leptomeningeal lymphomatosis, also known as “lymphomatous meningitis” (11, 12), angiotropic lymphoma (13, 14), and lymphomatous choroiditis (5, 6, 15, 16). If leptomeningeal lymphomatosis and lymphomatous choroiditis represent infiltrative patterns of neoplastic lymphocytes occurring in the leptomeninges or choroid plexuses, respectively (5), the angiotropic lymphoma is characterized by the proliferation of neoplastic lymphocytes within the lumen of small blood vessels (5). As for the spinal cord, lymphoma is reported as a solitary mass often growing in the extra-axial site, beyond the dura mater (extradural), or within it without any involvement of the spinal cord parenchyma (intradural-extramedullary). In the first case, it tends to extend into and through the adjacent bone and muscle tissues, rarely into the spinal cord parenchyma (6). On the contrary, lymphoma which develops as an intradural-extramedullary tumor, after replacing subdural and subarachnoid spaces, tends to infiltrate the adjacent spinal cord or nerve roots (5). Rarely, the spinal cord lymphoma develops as an intra-axial tumor (17). At last, feline lymphoma can also affect peripheral nerves (neurolymphomatosis) in form of a mononeuropathy, involving selected cranial or spinal nerves, or brachial plexopathy (18, 19). In a single case, feline neurolymphomatosis has been reported as an asymmetrical regional neuropathy involving brachial plexus, sciatic, and spinal nerve roots (20).

To define the immunophenotype and lymphoma subtypes is an essential step for further potential prognostic and therapeutic applications. For this purpose, immunohistochemistry, when flow cytometry is not in use, is strongly required. To date, studies concerning immunophenotype of feline nervous system lymphoma referred to specific anatomical patterns are still fragmentary since generally performed on single cases or small case cohorts. Similarly, few data are reported on the nervous system lymphoma grade of cats (7).

This study aimed to describe the specific localization and anatomical pattern of 24 feline lymphomas for which the immunophenotype was identified by immunohistochemistry investigations to support the potential specific correlation between subtypes and anatomical patterns.

## Methods

In total, twenty-four formalin-fixed and paraffin-embedded cases of feline lymphoma affecting the central and peripheral nervous system were used in this study. They were reported in the archive of the Neuropathology Laboratory of the University of Perugia (Italy) between 2007 and 2021 and consisted of 12 biopsies and 12 necropsy samples. Data regarding breed, sex, and age of the animals, and anatomical localization of the tumors are reported in Table 1. Histologically, the tumors were classified as small (1–1.5 RBC), intermedium (2 RBC), and large (>2 RBC) cell lymphoma, comparing the nucleus of the neoplastic lymphocytes to the diameter of a red blood cell (RBC). Tumors were histologically graded based on the mitotic count. According to what is established in the dog, this was defined by counting the number of mitotic figures in 10 fields at 400 × (2.37 mm^2^) in areas showing the highest replicative activity and then average determined; low grade = 0–5 mitoses; intermedium grade = 6–10 mitoses; high-grade = >10 mitoses (21).

**Table 1:** Signalment data, anatomical details of tumors, and lymphoma subtypes.

**N**.	**Breed**	**Sex**	**Age**	**Biopsy/necropsy**	**Anatomical side**	**Localization**	**Anatomical pattern**	**Lymphoma subtype**
1	ND	ND	12	Biopsy	Spinal cord	T8-T9	Extra-dural mass	DLBCL
2	DSH	MC	9	Biopsy	Brain	Frontotemporal cortex	Extra-axial mass	DLBCL
3	DSH	M	12	Necropsy	Spinal cord	C8-T3	Extra-dural mass	PTCL
4	DSH	MC	6	Necropsy	Brain	Parieto-occipital cortex	Intra-axial angiotropic	PTCL
5	DSH	FS	2	Biopsy	Spinal cord	T12-L2	Extra-dural mass	DLBCL
6	DSH	FS	2	Biopsy	Spinal cord	L7	Extra-dural mass	DLBCL
7	DSH	ND	11	Necropsy	Spinal root	Thoracic root	Neurolymphomatosis	LPL
8	DSH	FS	10	Necropsy	Spinal cord	Thoracic segment	Intra-axial mass	PTCL
9	ND	MC	11	Biopsy	Peripheral nerve	Peroneal nerve	Neurolymphomatosis	PTCL
10	DSH	MC	8	Necropsy	Peripheral nerve	Optic chiasm	Neurolymphomatosis	DRL
11	DSH	FS	4	Necropsy	Brain	Olfactory lobe	Intra-axial mass	DLBCL
12	DSH	F	1	Necropsy	Brain	Olfactory lobe	Intra-axial angiotropic	LPL
13	DSH	F	1	Biopsy	Brain	Olfactory lobe	Intra-axial mass	DLBCL
14	ND	F	12	Necropsy	Brain	Olfactory lobe frontal sinuses	Extra-axial mass	Not-RL
15	DSH	M	13	Biopsy	Brain	Hypothalamus	Leptomeningeal lymphomatosis	PTCL
16	Siamese	FS	14	Necropsy	Brain	Basal nuclei and thalamus	Intra-axial mass	Not-RL
17	ND	FS	11	Necropsy	Peripheral nerve	Optic chiasm	Neurolymphomatosis	DLBCL
18	DSH	MC	8	Biopsy	Spinal cord	T12	Extra-dural mass	DLBCL
19	ND	M	2	Biopsy	Brain and spinal cord	Cerebellum C1-C2	Extra-axial mass	DRL
20	DSH	M	1	Biopsy	Spinal cord	C4-C6	Extra-dural mass	DLBCL
21	DSH	F	15	Necropsy	Spinal cord	T1-L7	Leptomeningeal lymphomatosis	PTCL
22	DSH	MC	8	Necropsy	Brain, peripheral nerves, and eyes	Hypothalamus and Optic chiasm	Leptomeningeal lymphomatosis Neurolymphomatosis	DLBCL
23	DSH	MC	9	Biopsy	Spinal cord	T13-L2	Extra-dural mass	DBCL
24	DSH	MC	10	Biopsy	Spinal cord	C4	Extra-dural mass	DLBCL

ND, not defined; DSH, Domestic short hair; MC, castrated male; FS; neutered female; DLBCL, Diffuse large B cell lymphoma; DBCL, Diffuse B cell lymphoma; PTCL, Peripheral T cell lymphoma; LPL, lymphoplasmacytic lymphoma; DRL, double-reactive lymphoma; Not-RL, not-reactive lymphoma.

4 μm sections were used for immunohistochemistry (IHC) investigations. Immunolabeling was performed with the avidin-biotin-peroxidase complex (ABC) method using the following primary antibodies: rabbit polyclonal anti-human-CD20, mouse monoclonal anti-human-CD79a, rabbit polyclonal anti-human-CD3, mouse monoclonal anti-human-Paried Box 5 (PAX5), mouse monoclonal anti-human-multiple myeloma 1 (MUM-1), rabbit monoclonal anti-human-CD56, and rat monoclonal anti-human-CD44 (Table 2). After deparaffinization and rehydration, antigen retrieval was performed by microwave for 20 min in Tris-EDTA buffer solution (10 mmol/L Tris Base, 1 mmol/L EDTA, pH 9.0) for antibodies anti-CD3, CD79a, PAX5, MUM1, and in Tris-EDTA buffer solution (10 mmol/L Tris Base, 1 mmol/L EDTA, ph 6.0) for antibodies anti-CD44 and CD56. No retrieval was performed for the anti-CD20 antibody. Endogenous peroxidase was blocked using 3% hydrogen peroxide in water for 10 min at room temperature. Immunoreactivity was revealed by the avidin-biotin-peroxidase complex method (Abcam, Cambridge, UK) using 3-aminoetile-9-ethylcarbazole (AEC) substrate. Carazzi's hematoxylin was used as a counterstain. The feline lymph node was used as both, positive and negative control. In the latter case, slides were submitted to IHC in the same manner with the omission of the primary antibody. When present, normal B or T lymphocytes infiltrating the tumor were also used as an internal positive control. A semi-quantitative scoring system was used to assess the percentage of positive neoplastic cells: (–) no present; (+) <25% of sample; (++) 25–50% of sample; (+++) 50–75% of sample; (++++) >75% of sample.

**Table 2:** Primary antibodies and antigen retrieval methods used in immunohistochemical investigations.

**Antibody**		**Dilution**	**Source**	**Antigen retrieval**
CD20	Anti-human rabbit polyclonal	1:400	Thermo Fisher Scientific, Monza, IT	No retrieval
CD79a	Anti-human mouse monoclonal, sc-53208	1:100	Santa Cruz Biotechnology, Dallas	Tris/EDTA buffer, pH 9.0
CD3	Anti-human rabbit polyclonal	1:200	Dako, Denmark	Tris/EDTA buffer, pH 9.0
PAX5	Anti-human mouse monoclonal, 24/PAX-5	1:20	BD Biosciences, New York	Tris/EDTA buffer, pH 9.0
MUM-1	Anti-human mouse monoclonal, MUM1p	1:75	Dako, Denmark	Tris/EDTA buffer, pH 9.0
CD56	Anti-human Rabbit monoclonal, RCD56	1:150	Zytomed, Berlin	Tris/EDTA buffer, pH 6.0
CD44	Anti-human Rat monoclonal, IM7	1:100	Thermo Fischer Scientific, Monza, IT	Tris/EDTA buffer, pH 6.0

## Results

**Figure 1:**
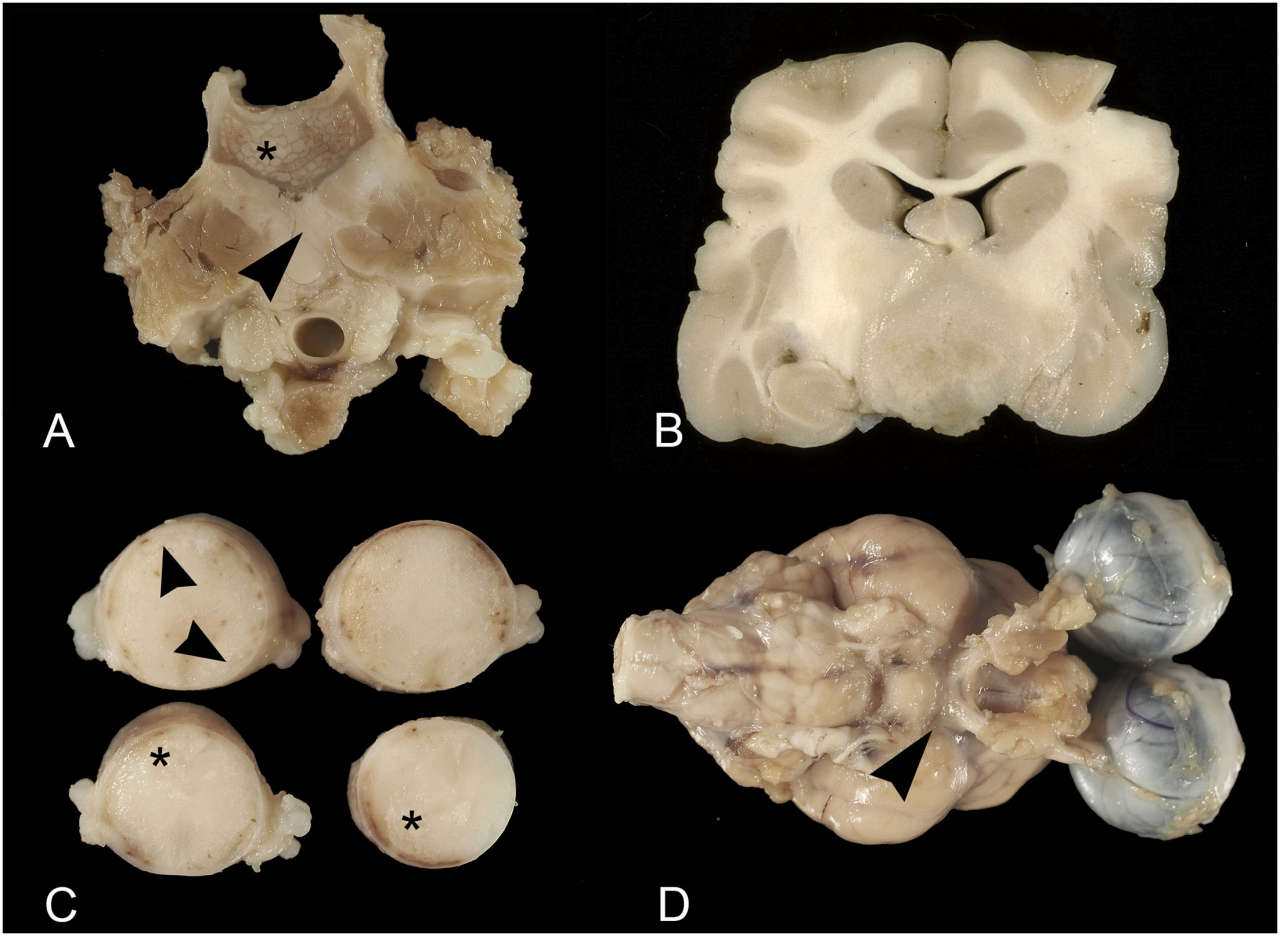
Feline lymphoma of the nervous system. **(A)** Extra-axial lymphoma. Vertebral column at the level of L1. The vertebral body is partially replaced by lymphomatous tissue (asterisk) infiltrating the adjacent iliopsoas muscles (arrowhead); **(B)** Intra-axial lymphoma. Transverse brain section at the level of the rostral diencephalon. Intra-axial mass of whitish rather firm tissue, involving the brain base; **(C)** Leptomeningeal lymphomatosis. Thoracic spinal cord segments. Note the irregular leptomeningeal profile (arrowheads) and subpial parenchyma with large softening areas (asterisks); **(D)** Neurolymphomatosis and leptomeningeal lymphomatosis. Thickening of the optic nerves emerging from the optic chiasm and irregular leptomeningeal profile (arrowhead).

The affected animals were from 1 to 15-year-old (mean age = 8 years). In 17 cases, they were more than 5 years. Domestic short hair cats were the most represented (18 cases). In total, 10 animals were females and 12 were males. In two cases the sex was not reported. Ten tumors affected the spinal cord, eight the brain, four the peripheral nerves; one involved both the brain and the spinal cord, and one both the brain and the optic nerves (Table 1). All the tumors were primary lymphomas, except for two cases (case 3 and case 14) occurring in association with the involvement of additional systems. In 16 cases lymphoma developed as a mass, in 11 cases occurring with an extra-axial pattern (eight spinal cords, two brains, one both the brain and spinal cord), (Figure 1A), and in the remaining six cases (five brains and one spinal cord) with intra-axial pattern (Figure 1B). Extra-axial lymphomas of the spinal cord were all at the extra-dural site, except one case. In three cases, lymphoma developed as leptomeningeal lymphomatosis (Figure 1C) and in five cases, it diffusely involved peripheral nerves (neurolymphomatosis). In total, three cases of neurolymphomatosis affected the optic chiasm (Figure 1D). One case expressed both brain leptomeningeal lymphomatosis and neurolymphomatosis (optic chiasm) patterns. Two intra-axial lymphomas of the brain showed the angiotropic pattern (Table 1). In all the cases growing as a mass, the tumor consisted of a whitish and soft translucent tissue, sometimes with small foci of yellow necrosis. Leptomeningeal lymphomatosis occurred as an irregular thickening of the meningeal profile with softening of the adjacent nervous parenchyma. In a similar manner, neurolymphomatosis appeared as a more or less irregular enlargement of the involved nerves.

At optic microscopy, in the mass lesions, the neoplastic cells tended to arrange in sheets sustained by thin fibrovascular stroma, merging in solid areas. In the brain, infiltration of the adjacent parenchyma often occurred with a perivascular pattern. In the spinal cord, the neoplastic tissue tended to involve the spinal nerve roots, the extra-dural adipose tissue, and/or the adjacent vertebral body along with the paravertebral soft tissues. Often, the neighboring spinal cord white matter showed changes secondary to the tumor compression, ranging from degeneration, with spongiosis and spheroids, to necrosis.

Leptomeningeal lymphomatosis was characterized by diffuse neoplastic infiltration of the subarachnoid space with a confluent perivascular pattern, also involving the adjacent nervous tissue. In neurolymphomatosis, sheets of neoplastic cells infiltrated the endo- and perineurial sheaths. Axonal enlargement and degeneration were also observed. At the optic chiasm, the adjacent nervous tissue was involved through a perivascular pattern as well.

**Figure 2:**
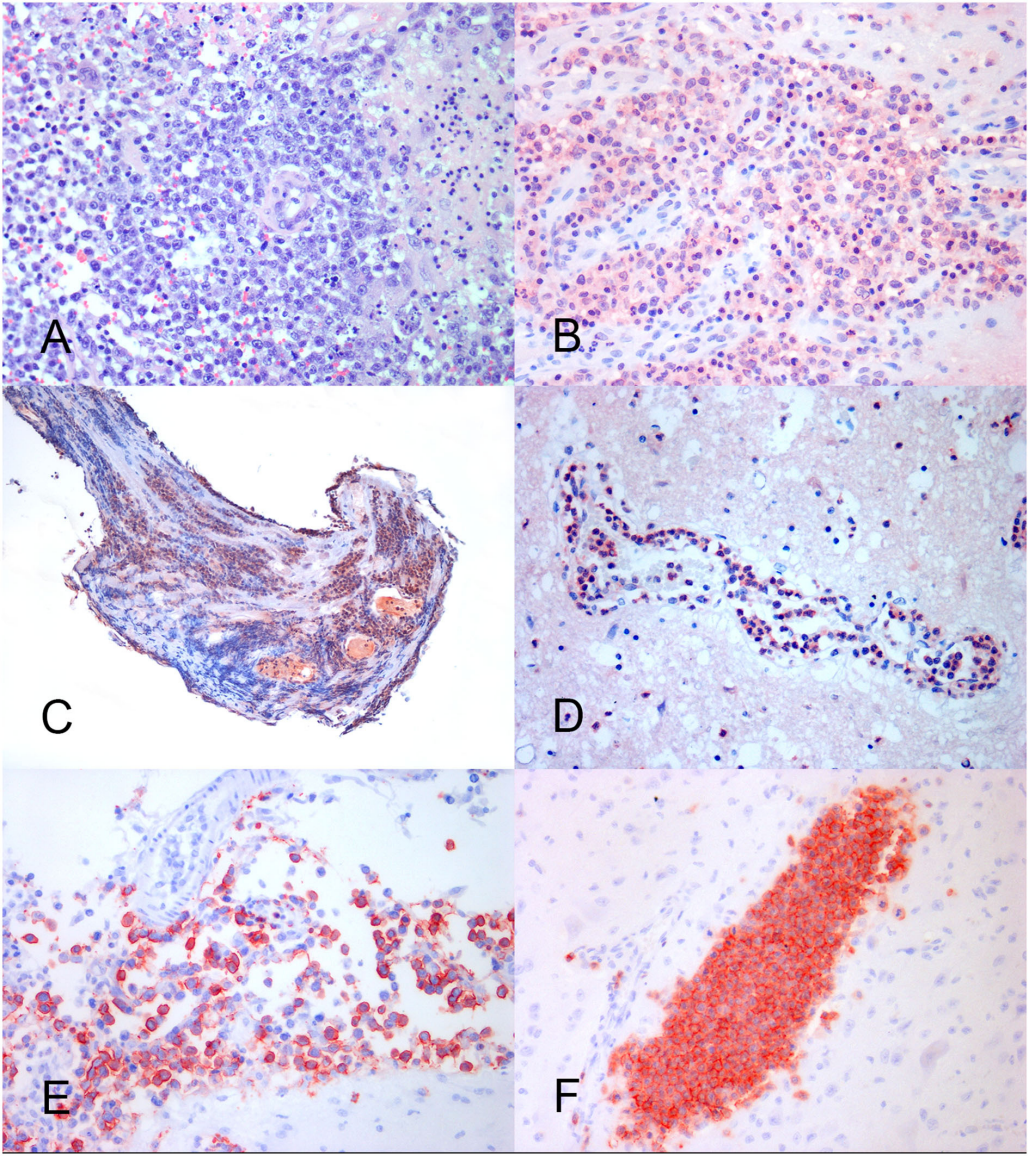
Feline lymphoma of the nervous system. **(A)** Neoplastic large lymphocytes with the round nucleus and prominent nucleolus, arranged in a perivascular pattern. HE, × 40. **(B)** B cell brain lymphoma showing an area of consistent MUM-1 positive nuclear and cytoplasmic immunoreaction. IHC, × 40. **(C)** Spinal nerve root. Lymphoplasmacytic lymphoma with a prevalence of MUM-1 positive neoplastic cells. IHC, × 20. **(D)** Brain angiotropic lymphoma showing marked MUM-1 positive cell reaction into the blood vessel lumen. IHC, × 40. **(E)** B cell brain leptomeningeal lymphomatosis. The subarachnoid space is crowed with CD20-positive neoplastic lymphocytes. IHC, × 40. **(F)** Brain angiotropic T cell lymphoma. A parenchymal blood vessel is filled with CD3-positive neoplastic cells. IHC × 40.

Neoplastic tissue was densely packed by the monomorphic population of round to polygonal cells, with a hyperchromatic nucleus with dispersed chromatin, from absent to prominent nucleolus, and scant eosinophilic cytoplasm confined to a perinuclear rim. In total, nineteen tumors were consistent with large-cell lymphomas (LCL) (Figure 2A), while the remaining five tumors were consistent with intermediate-cell lymphomas (ICL). A total of nineteen lymphomas were of low grade, three of intermedium grade, and two of high grade (Table 3).

**Table 3:** Immunohistochemical findings and final diagnosis.

**N**.	**Diagnosis**	**Cell size**	**Grade**	**CD3**	**CD20**	**CD79a**	**PAX-5**	**MUM-1**	**CD56**	**CD44**
1	DLBCL	LCL	Intermediate	+	+++	++++	++++	++	–	+
2	DLBCL	LCL	Low	+	++++	++++	++++	++++	–	+
3	PTCL	ICL	Low	+++	–	–	–	++	–	–
4	PTCL	LCL	High	++++	–	–	–	–	–	–
5	DLBCL	LCL	Low	+	+++	++++	++++	++	–	+
6	DLBCL	LCL	Low	+	++++	–	++++	+	–	–
7	LPL	LCL	Low	+	+	–	+	++++	–	–
8	PTCL	LCL	High	++++	+	–	+	+	–	++
9	PTCL	LCL	Low	++++	–	–	–	–	–	+
10	DRL	ICL	Low	+	+	+	+	+	–	+
11	DLBCL	LCL	Low	+	++++	+++	+++	++++	–	+++
12	LPL	ICL	Low	–	–	–	–	++++	–	+
13	DLBCL	LCL	Low	+	+++	++++	++++	+	–	+
14	Not–RL	LCL	Low	–	–	–	–	–	–	+
15	PTCL	ICL	Low	++++	–	–	–	–	–	+
16	Not–RL	LCL	Low	–	–	–	–	–	–	–
17	DLBCL	LCL	Low	–	++++	++++	++++	–	–	++++
18	DLBCL	LCL	Low	+	++++	++++	++++	+	–	+
19	DRL	LCL	Intermediate	+	+	–	+	+	+	+
20	DLBCL	LCL	Low	–	+	++++	++++	–	–	–
21	PTCL	LCL	low	++++	–	–	–	+++	–	–
22	DLBCL	LCL	Intermediate	–	–	+++	–	–	–	+
23	DBCL	ICL	Low	–	+++	–	–	++++	–	–
24	DLBCL	LCL	Low	+	++	++	++	+	–	+

(–), negative; (+), <25%; (++), 25–50%; (+++), 50–75%; (++++), >75%.

DLBCL, Diffuse large B–cell lymphoma; DBCL, Diffuse B–cell lymphoma; PTCL, Peripheral T cell lymphoma; DRL, double-reactive lymphoma; LPL, lymphoplasmacytic lymphoma; Not-RL, not-reactive lymphoma; LCL, large cell-lymphoma; ICL, intermediate cell-lymphoma.

At immunohistochemistry, the prevalent cell immunoreactivity for B cell markers (CD20, CD79a, and PAX5) or for the T cell marker (CD3) was considered diagnostic for B cell (BCL) and T cell lymphoma (TCL), respectively. Tumors expressing a similar percentage of cell immunoreactivity for B cell and T cell markers were considered double-reactive lymphomas (DRL). The MUM-1 reaction associated with prevalent B cell marker or PAX5 immuno-expression was adopted to indicate B cell lymphoma (Figure 2B). The MUM-1 associated with prevalent CD3-immunoreaction, in the absence of B cell marker reaction, was considered diagnostic for T cell lymphoma. Prevalent MUM-1 immunoreaction alone was considered diagnostic for plasmacytoid differentiation (lymphoplasmacytic lymphoma = LPL). A low number of immunoreactive cells for B cell markers vs. a prevalence of the immunoreactive cells for T cell markers, and vice versa, were considered consistent with reactive lymphocytes in T cell or B cell lymphoma, respectively (Table 3).

Based on the results, 12 tumors consisted of diffuse B cell lymphomas (DBCL) including 11 LCLs (DLBCL) and 1 ICL. On the contrary, six tumors, including four LCLs and two ICLs, consisted in TCL which were considered no more specific than a peripheral T cell lymphoma (PTCL). One LCL and one ICL were DRL, while two cases of LCL were negative for all the tested markers. In total, two tumors were considered as LPL. They consisted of a case of neurolymphomatosis (Figure 2C) and a case of brain angiotropic lymphoma (Figure 2D), respectively (Table 3). B cell lymphoma affected animals of 6.4 years in mean age, while T cell lymphoma affected older animals (11.1 mean age). Out of eleven extra-axial tumors, eight consisted of B-cell lymphomas.

Neurolymphomatosis expressed B cell immunophenotype in two cases; one case was a PTCL, one LPL, and the remaining was consistent with a DRL. Two out of the three cases of leptomeningeal lymphomatosis expressed T cell immunophenotype, while the remaining expressed B cell immunophenotype (Figure 2E). First angiotropic pattern was of T cell phenotype (Figure 2F) while the second consisted in a LPL. Two lymphomas localized at the optic chiasm expressed B cell phenotype. The remaining was a DRL. PAX5 was expressed in 10 out of 12 DBCL (Table 3).

All the cases were CD56-negative, except for a case consisting of a DRL. The CD44-expression was found in 16 cases, consisting of seven extra-axial and four intra-axial tumors, four neurolymphomatosis, and one leptomeningeal lymphomatosis (Table 3).

## Discussion

In this study, 24 feline lymphomas of the nervous system growing with different anatomical patterns were submitted to phenotyping investigation by immunohistochemistry using CD20, CD79a, PAX5, and MUM-1 as B cell markers, CD3 as a T cell marker, CD56 as a marker for NK cells or T cells (19), and CD44 to mark tumor cell interaction with host tissue cells and extracellular-matrix (22). Diagnosis of lymphoma was based on histological findings that mainly addressed to monomorphic population of lymphoid cells which was considered not consistent with any of the potential feline inflammatory diseases of the nervous system. About 71% of animals were more than 5 years and, and although the FeLV test was not reported for them, based on the animal age (only six animals <3 years) we can hypothesize that FeLV had been uninfluential in causing the neoplastic disease (2, 3, 6). As recently reported (6), B cell lymphoma affected animals younger than those suffering from T cell lymphoma. B cell phenotype was the most represented (50%) followed by T cell lymphoma (25%), mimicking what was reported in dogs (23). Significant differences were not observed between the sexes. The domestic short-haired cat was the most represented, reflecting the high diffusion of this breed in the Italian feline population.

In this study, the spinal cord was the preferential site for lymphoma (45.8% of cases) compared to the brain (41.6% of cases) (6, 7). However, for the brain lymphoma, the percentage tended to consistently decrease if related to all the brain tumors (69 cases) diagnosed in our lab in the same period (14.4%). For both the brain and spinal cord in our database, the first tumor remains the meningioma probably since our neuropathology laboratory is a reference center in studying animal meningioma in Italy. However, these data strongly confirm lymphoma to be significantly representative of central nervous system tumors in the cat. As opposed to any expectation, in this study 22 cases (91.6%) consisted of primary lymphomas. For the biopsy cases, the exclusion of more organs and systems involvement by the neoplasia was made clinically, for the necropsy cases at post-mortem investigations. It might be feasible that the increase of specialized neurology services in veterinary medicine occurred in the last years have contributed to the identification and/or surgical treatment of primary lymphoma as compared with the past, when this form was considered much rarer (24). Although a larger cohort of cases is necessary to confirm such an apparent change of course, this result appears to be consistently contrary to that reported in the dog as well (23). A cohort of ten feline primary nervous system lymphomas accounting for 27% of nervous system lymphomas recently described supports this new perspective should not be ignored in the clinical approach (7).

As for the anatomical localization, feline lymphoma of the spinal cord mainly developed as extra-axial mass (81.8%), while in the brain it was mainly an intra-axial tumor (62.5%) confirming previous studies in cats (6, 8, 9, 25). We also confirmed that the majority of the spinal cord lymphomas occurred in the thoracolumbar region (6, 9, 17), and the cerebral cortex and diencephalon as the anatomical sites most commonly affected in the brain (8). All the brain lymphomas, except one, developed in the cranial fossa preferring the olfactory-frontal lobe, followed by the diencephalon. Most often, they occurred as a localized non-encapsulated mass. The only lymphoma of the posterior fossa involved the cerebellar cortex as an extra-axial mass that extended up to the C2 spinal cord segment. To date, the literature reports very few cases of feline cerebellar lymphomas occurring as intraparenchymal tumors (24) or not (26). As for the angiotropic lymphomas, both the cases of this study affected the brain confirming for this site the few cases reported in the literature (13, 14). The most numerous cases reported in dogs (27) seem to confirm that in pets the spinal cord and peripheral nerves are very rarely affected by angiotropic lymphoma (28).

In this study, two out of three cases of leptomeningeal lymphomatosis were observed in the brain. However, to conclude this anatomical pattern of lymphoma most often develops as an intracranial tumor is rather arbitrary because of the low number of reported cases. Nowadays, the literature reports a single case of diffuse infiltration of spinal cord leptomeninges by atypical lymphocytes in the cat in association with eosinophilic meningomyelitis (29) compared with the four cases of brain leptomeningeal lymphomatosis occurred as a secondary multicentric-form derived (6). On the contrary, we believe the optic chiasm is a preferential site for the neurolymphomatosis. This data, not previously reported, is worthy of specific attention in the future to be confirmed. The lymphomatosis cerebri and lymphomatous choroiditis were not observed in this study at all.

Immunohistochemistry is an essential step in classifying and phenotyping lymphoma, although results are unable to avoid misinterpretations. The CD20, CD79a, and PAX5 are commonly used to identify B cell lymphomas, while CD3 is a specific marker for T cell lymphoma. B cell lymphoma can be reactive to CD20, and CD79a or PAX5. The different combination among these three markers seems to suggest different stages of differentiation for B lymphocytes. In fact, CD20 and CD79a are expressed at all the stages of B cell differentiation; however, CD20 is not expressed in mature plasma cells at all (23). Based on this, in the case of LPL herein reported the few cells expressing CD20 were considered reactive cells. On the contrary, PAX5 is a pan B cell marker in mammalian species (30) expressed in the early stages of B cell differentiation, being completely lost in mature cells (23). The MUM-1 is a transcriptional factor expressed in terminally differentiated B-lymphocytes that is plasma cells (31). However, its expression has also been reported in reactive T lymphocytes (31, 32). Cases 10 and 19 showed immunoreactions for both B cell markers, MUM-1 included, and T cell markers, although for any of them prevalent. Based on these results they were classified as double-reactive lymphomas (DRL). Cases 14 and 16 were negative for all the tested markers. Considering they expressed internal control, we can assume to be consistent with non-B non-T lymphomas, instead of false negative cases associated with an excess of fixation or autolysis.

The extra-axial lymphomas which occurred as a mass mainly consisted of B cell lymphomas (8/11) of low grade, while the leptomeningeal lymphomatosis mainly expressed T cell immunophenotype of low grade (2/3) (11). Unfortunately, the literature lacks further consistent data about immunophenotype for this anatomical pattern in the cat (6). On the contrary, this result sounds in contrast with those reported in dogs (23), although it is not clear if the meningeal pattern considered by the authors is really consistent with proper leptomeningeal lymphomatosis.

The intra-axial lymphomas were B cell and T cell lymphomas supporting the perception that contrary to dogs (23), feline intra-parenchymal lymphomas do not express preferential immunophenotype (5, 24, 29, 33). The two angiotropic lymphomas were a T cell lymphoma and an LPL, respectively. In the cat, to date, a case of angiotropic lymphoma of T cell origin (13) and a case of mixed lineage antigen expression (14) have been reported. In dogs, lymphoma with angiotropism has been reported with T cell phenotype as the most prevalent (23) or not (27, 34). To our knowledge, angiotropic LPL of the nervous system has not been reported yet in cats or in dogs. For this case, multiple myeloma was clinically and at necropsy excluded. As for the neurolymphomatosis pattern, it remains the most common secondary tumor of peripheral nerves in cats albeit feline lymphoma rarely involves the peripheral nervous system (35). Neurolymphomatosis has been also reported as a primary and isolated event preferentially involving the brachial plexus (18, 19, 35). The studies of immunophenotyping support that B cell lineage is the most expressed in this anatomical pattern (19), contrary to what is reported in dogs (23). In this study, neurolymphomatosis did not express a prevalent immunophenotype. The most significant result we believe is worth to be emphasized is a case of lymphoplasmacytic neurolymphomatosis. To our knowledge, this is the first LPL of the peripheral nerves reported in the cat.

The CD56, also called neuronal cell adhesion molecule, is expressed on cells of the central nervous system, natural killer (NK) cells, and subsets of T cells as well (36). The CD56 is also reported to be abnormally expressed by B cells when they undergo neoplastic transformation (19). In this study, it was expressed on neoplastic cells in only one case, along with B and T cell markers. Although the tumor was considered a DRL with CD56 aberrant expression, the single identification of this finding raises difficulties in the possible suggestion of the cell lineage of the origin or the aggressive behavior of the tumor (36).

The CD44 is an extensively expressed cell surface glycoprotein of 80–200 kDa involved in cell–cell adhesion (22) and cell-extracellular matrix (ECM) interaction (22) besides lymphocyte activation (16). Many CD44 isoforms have been detected supporting that the effects of its expression in tumors can be produced by several mechanisms (22). In the last years, CD44-expression has been investigated in canine cerebral angiotropic lymphoma with positive results expressed by neoplastic and endothelial cells (27). In this study, CD44 was expressed in all the anatomical patterns of nervous system lymphoma supporting for them a common malignant potential. However, the exact functional role of this molecule should be considered with caution when investigated with immunohistochemistry.

In conclusion, this study reports a prevalent primary feline lymphoma of the nervous system mainly expressed as a spinal cord extra-axial mass. The leptomeningeal lymphomatosis mainly occurred in the brain while the optic chiasm resulted to be the preferential site of development for neurolymphomatosis. The B cell lymphomas seem to affect a class of age lower than T cell lymphoma. Moreover, B cell lymphoma has been detected as the most expressed phenotype in nervous system tumors targeting extra-axial tumors, and interestingly, the optic chiasm lymphoma. On the contrary, the T cell phenotype seemed to target leptomeningeal lymphomatosis. For the first time plasmacytoid differentiation was found for angiotropic lymphoma and neurolymphomatosis.

Although lymphoma represents in the cat the main suspicion among the differential diagnoses of the central and peripheral nervous system diseases, the final diagnosis of lymphoma needs careful histological examination also to support suggestive magnetic resonance and cerebrospinal fluid findings. For the definitive diagnosis, when flow cytometry is not in use, immunohistochemistry investigations become irrevocable. Therefore, to improve information about the immunophenotype of feline lymphoma of the nervous system should be strongly required in the diagnostic workup and clinical approach to this tumor especially when its primary origin is confirmed.

## Data availability statement

The original contributions presented in the study are included in the article/supplementary material, further inquiries can be directed to the corresponding author/s.

## Author contributions

MM, AD, and GG contributed to conception and design of the study. MM analyzed the data and wrote the first draft of the manuscript. AD and GG collected and organized the data. All authors contributed to the article and approved the submitted version.

## Funding

This study was supported by the University of Perugia (IT) through the Basic Research funds 2017–2019.

## Conflict of interest

The authors declare that the research was conducted in the absence of any commercial or financial relationships that could be construed as a potential conflict of interest.

## Publisher's note

All claims expressed in this article are solely those of the authors and do not necessarily represent those of their affiliated organizations, or those of the publisher, the editors and the reviewers. Any product that may be evaluated in this article, or claim that may be made by its manufacturer, is not guaranteed or endorsed by the publisher.
